# Leber’s Hereditary Optic Neuropathy: the roles of mitochondrial transfer RNA variants

**DOI:** 10.7717/peerj.10651

**Published:** 2021-01-18

**Authors:** Yu Ding, Guangchao Zhuo, Qinxian Guo, Meiya Li

**Affiliations:** 1 Central laboratory, Hangzhou First People’s Hospital, Hangzhou, Zhejiang, China; 2 Academy of Chinese Medical Sciences, Zhejiang Chinese Medical University, Hangzhou, Zhejiang, China

**Keywords:** mt-tRNA, Variants, LHON, tRNA metabolism, OXPHOS

## Abstract

Leber’s Hereditary Optic Neuropathy (LHON) was a common maternally inherited disease causing severe and permanent visual loss which mostly affects males. Three primary mitochondrial DNA (mtDNA) mutations, *ND1* 3460G>A,* ND4* 11778G>A and *ND6* 14484T>C, which affect genes encoding respiratory chain complex I subunit, are responsible for >90% of LHON cases worldwide. Families with maternally transmitted LHON show incomplete penetrance with a male preponderance for visual loss, suggesting the involvement of secondary mtDNA variants and other modifying factors. In particular, variants in mitochondrial tRNA (mt-tRNA) are important risk factors for LHON. These variants decreased the tRNA stability, prevent tRNA aminoacylation, influence the post-transcriptionalmodification and affect tRNA maturation. Failure of mt-tRNA metabolism subsequently impairs protein synthesis and expression, folding, and function of oxidative phosphorylation (OXPHOS) enzymes, which aggravates mitochondrial dysfunction that is involved in the progression and pathogenesis of LHON. This review summarizes the recent advances in our understanding of mt-tRNA biology and function, as well as the reported LHON-related mt-tRNA second variants; it also discusses the molecular mechanism behind the involvement of these variants in LHON.

## Introduction

Leber’s Hereditary Optic Neuropathy (LHON) is named after Theodore Leber, a German ophthalmologist who first described the defining clinical features of this disorder in 1871. LHON is the commonest maternally inherited eye diseases, which typically affects young adults, with most of patients being males (Sandbach & Newman, 2001; Man, Turnbull & Chinnery, 2002). Vision loss from LHON results from selective degeneration of retinal ganglion cells (RGCs) (*Carelli et al., 2009*). Loss of RGCs occurs in around 50% of male and but only in approximately 10%∼15% of female patients. It causes adult-onset progressive and painless visual loss which begins in only one eye, but usually manifestes in the other eye within a few weeks. Eventually, visual acuity in both eyes deteriorated to 20/200 or worse. Moreover, LHON patients may exhibit abnormal symptoms, including movement disorders, dystonia or multiple sclerosis like symptoms, which pose a significant challenge for clinicians (Yu-Wai-Man, Griffiths & Chinnery, 2011; Jia et al., 2006).

The prevalence of LHON has been well established in Northern European populations, with figures ranging from one in 30,000 to one in 50,000 (Man, Turnbull & Chinnery, 2002; Rosenberg et al., 2016; Yu-Wai-Man et al., 2003), and one in 1,000,000 in Japanese population according to a recent survey (*Ueda et al., 2017*). Clinically, over 90% of LHON cases are caused by one of three mtDNA missense mutations in genes encoding subunits of NADH dehydrogenase (*ND*): *ND1* 3460G>A, *ND4* 11778G>A and *ND6* 14484T>C (*Wallace et al., 1988; Catarino et al., 2017; Huoponen et al., 1991*). Although the genetic basis of LHON was remains unclear, it has become apparent that mitochondrial dysfunction caused by mtDNA mutations is the molecular basis of this disease. mt-tRNA genes are also highly susceptible to point mutations, which are primary causes of mitochondrial dysfunction (*Scaglia & Wong, 2008*). It is thus possible that mt-tRNA variants also play important roles in the phenotypic manifestation of LHON-associated primary mutations. In this review, we cover the basic aspects of mitochondrial biology and genetics, as well as mt-tRNA maturation, and summarize the mt-tRNA variants that have been reported to be associated with LHON.

## Review Methodology

We carried out a search in PubMed Central (http://www.ncbi.nlm.nih.gov) and other public domains with the following keywords: “mitochondrial biology”, “mtDNA genetics”, “mt-tRNA function”, “mt-tRNA maturation”, “mt-tRNA end processing”, “mt-tRNA modification”, “mt-tRNA variants and LHON” (last search update on October 8, 2020). The “OR” and “AND” terms were used for the various searches. We excluded studies if the crucial data were not reported in the original papers, or if there was a very high likehood of inaccurate reporting.

To investigate the candidate pathogenic mt-tRNA variants, the Mamit-tRNA database (http://mamit-tRNA.u-strasbg.fr) was used to locate the positions of the mt-tRNA variants, as well as the cloverleaf structure of tRNAs (Pütz et al., 2007). Additionally, the conversion of nucleotide numbering in human mt-tRNA genes was based on the criteria proposed by Andrews et al. (1999). The conservation index (CI) of each reported mt-tRNA variant was analyzed by the ClustalW program (http://www.ebi.ac.uk/Tools/msa/clustalw2/) (*Hall, 2013*).

### Mitochondrial biology and genetics

Mitochondria originated from within the bacterial phylum *α*-Proteobacteria and became established via an endosymbiotic event (*Lane & Martin, 2010*). Mitochondria are critical organelles that perform a remarkably diverse set of cellular functions. The most important of these is the generation of ATP via OXPHOS, but mitochondria also play critical roles in the regulation of apoptosis, maintenance of cellular redox homeostasis and intracellular calcium signaling (*Duchen, 2004; Tait & Green, 2010; Sena & Chandel, 2012; Rizzuto et al., 2012*).

**Figure 1:**
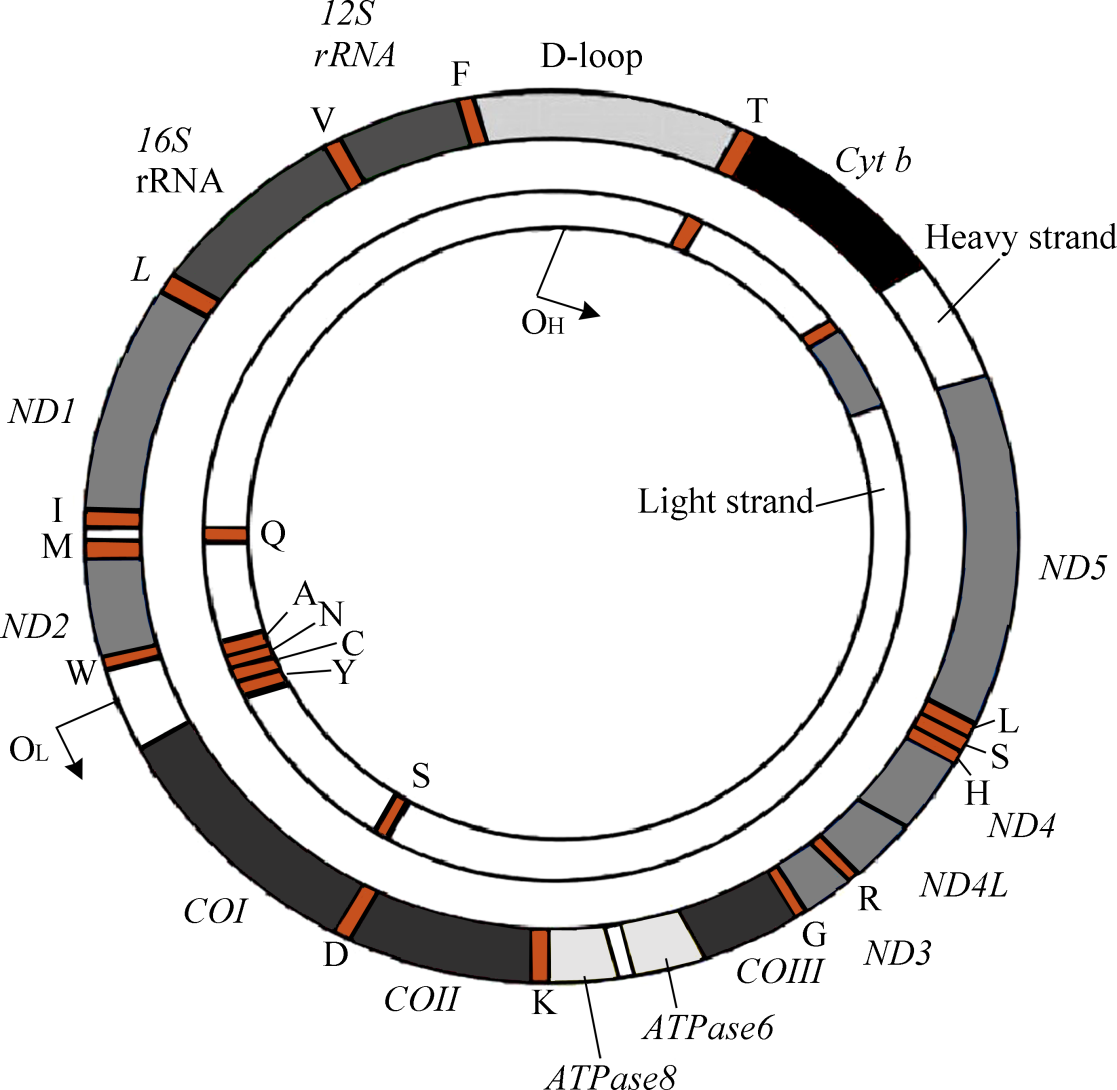
The genetic map of human mitogenome, which is a circular, double strand DNA.

The human mitochondrial genomes (mitogenomes) are circular, 16,569-bp in length, and contain 37 genes encoding 13 proteins required for OXPHOS and the electron transport chain (ETC) (Fig. 1) (*Andrews et al., 1999*). mtDNA also encodes RNAs, which are involved in the translation of ETC proteins (*Luo et al., 2018*). Owing to its location within the mitochondrial matrix, lack of protective histone wrapping, as well as a comparatively limited repair mechanism, mtDNA is more vulnerable to oxidative modifications which accumulate over time (Yakes & Van Houten, 1997). Indeed, it has been shown that mtDNA has a significantly higher mutation rate than nuclear DNA (*Neckelmann et al., 1987*).

Unlike nuclear DNA, in which there are only two copies of each gene per cell, thousands of copies of mtDNA are presented in every cell. Typically, individuals harbor only one mtDNA genotype, and all mitogenomes are genetically identical, a condition called homoplasmy. This contrasts with heteroplasmy, which involves the presence of a mixture of mutant and wild-type mtDNA genomes within a cell. Through somatic mutagenesis and ongoing replication of mtDNA, mutations can clonally expand through either random drift or selective processes, and become present at varying proportions or degrees of heteroplasmy with cells (*Elson et al., 2001*). Among families affected by LHON, 85%–90% of carriers are homoplasmic for mtDNA mutation. However, some studies have indicated that mtDNA heteroplasmy may be a factor determining the penetrance of LHON *(Li et al., 2019; Finsterer & Zarrouk-Mahjoub, 2018)*). In certain families, rapid segregation of the mitochondrial genotype toward mutant-type homoplasmy of either 11778G>A (*Bolhuis et al., 1990*) or 3460G>A mutation in blood (*Black et al., 1996*) has been shown to be associated with the development of LHON in later generations. It has been suggested that the risk of disease conversion is low if the mutational load is below the threshold of 60% (Chinnery et al., 2001). Although it is not possible to accurately predict whether an LHON carrier will eventually lose vision, individuals can be counseled based on the two major identifiable risk factors in this disorder: age and sex.

### Three LHON-associated primary mutations

The majority of patients with LHON harbor one of three primary mtDNA point mutations: 3460G>A (Howell et al., 1991; Huoponen et al., 1991), 11778G>A (*Wallace et al., 1988*), and 14484T>C (*Johns, Neufeld & Park, 1992; Mackey & Howell, 1992*). They are found exclusively in families affected by LHON and never in control subjects. The G to A transition at position 11778 converts a conserved arginine to histidine, has been associated with poor visual outcome and prognosis (*Newman, Lott & Wallace, 1991*). Meanwhile, the 3460G>A mutation causes the alteration of a highly conserved alanine to threonine, which is present in around 15% of LHON families (Howell, et al. 1991). Moreover, the T to C transition at nucleotide 14484 in *ND6* (methionine to valine) has been shown to be tightly linked to the LHON phenotype (*Johns, Neufeld & Park, 1992*). Interestingly, younger age at onset (<15 years) and mutation type appear to dictate visual outcome; patients with the 14484T>C mutation have a better visual prognosis with 60% attaining some visual improvement compared with only 5% of those carrying the 11778G>A mutation.

The incomplete penetrance, high male to female ratio, and existence of LHON plus cases strongly suggest the involvement of modifying factors such as genetic or environmental ones (Tońska, Kodroń & Bartnik, 2010; Caporali et al., 2017). In particular, genetic factors such as mt-tRNA variants can play active roles in the phenotypic manifestation of LHON-associated primary mutations.

### Nuclear genes

Although the mitochondrial proteome consists of over 1000 proteins, only 14 of them are encoded by mtDNA. Thus, the nuclear genome encodes >90% of peptides involved in the OXPHOS system (*Leigh-Brown, Enriquez & Odom, 2010*). Moreover, incomplete penetrance and male bias in patients with LHON suggest that an X-linked modified gene is necessary for the disease expression (*Bu & Rotter, 1991*). A recent genome-wide study of 1281 Chinese probands with LHON identified a novel LHON susceptibility allele (c.157C>T, p. Arg53Trp) in the *PRICKLE3* gene, which links to ATPase biogenesis manifested LHON (*Yu et al., 2020*). Moreover, a missense mutation in *YARS2* (c.572G>T, p. Gly191Val) was shown to interact with the 11778G>A mutation to cause visual failure (Jiang et al., 2016).

### mt-tRNA structure and function

mt-tRNA is a short, non-coding RNA that constitutes approximately 4∼10% of all cellular RNAs (*Kirchner & Ignatova, 2015*). In fact, most mt-tRNAs from all domains of life have a highly conserved cloverleaf structure, consisting of an acceptor arm, D-arm, anticodon stem, variable region, and TψC loop, with an average length of 73 nucleotides (nts). However, mt-tRNA genes encode transcripts that show considerable deviation of this standard, having a reduced D-arm or TψC loop or even completely lacking one of these elements (e.g., tRNA^Ser(AGY )^), resulting in tRNAs as small as 66 nts (*Hanada et al., 2001*). In addition, mt-tRNA^Ser(UCN)^ has several distinct structural features, including only one base (A9) between the acceptor arm and D-arm, a short D-loop, a variable region, and an extended anticodon stem with 6-bp (*Watanabe et al., 1994*).

As adapter molecules to convert the information stored in amino acid (AA) sequences, tRNAs play a central role in protein synthesis (*Stowe & Camara, 2009*). Although tRNAs comprise only around 10% of the total coding capacity of the mitogenomes , more than half of mtDNA mutations causing diseases are located in mt-tRNA genes (https://www.mitomap.org/MITOMAP) (*Taanman, 1999*), emphasizing the importance of tRNAs for mitochondrial function.

### tRNA end processing

**Figure 2:**
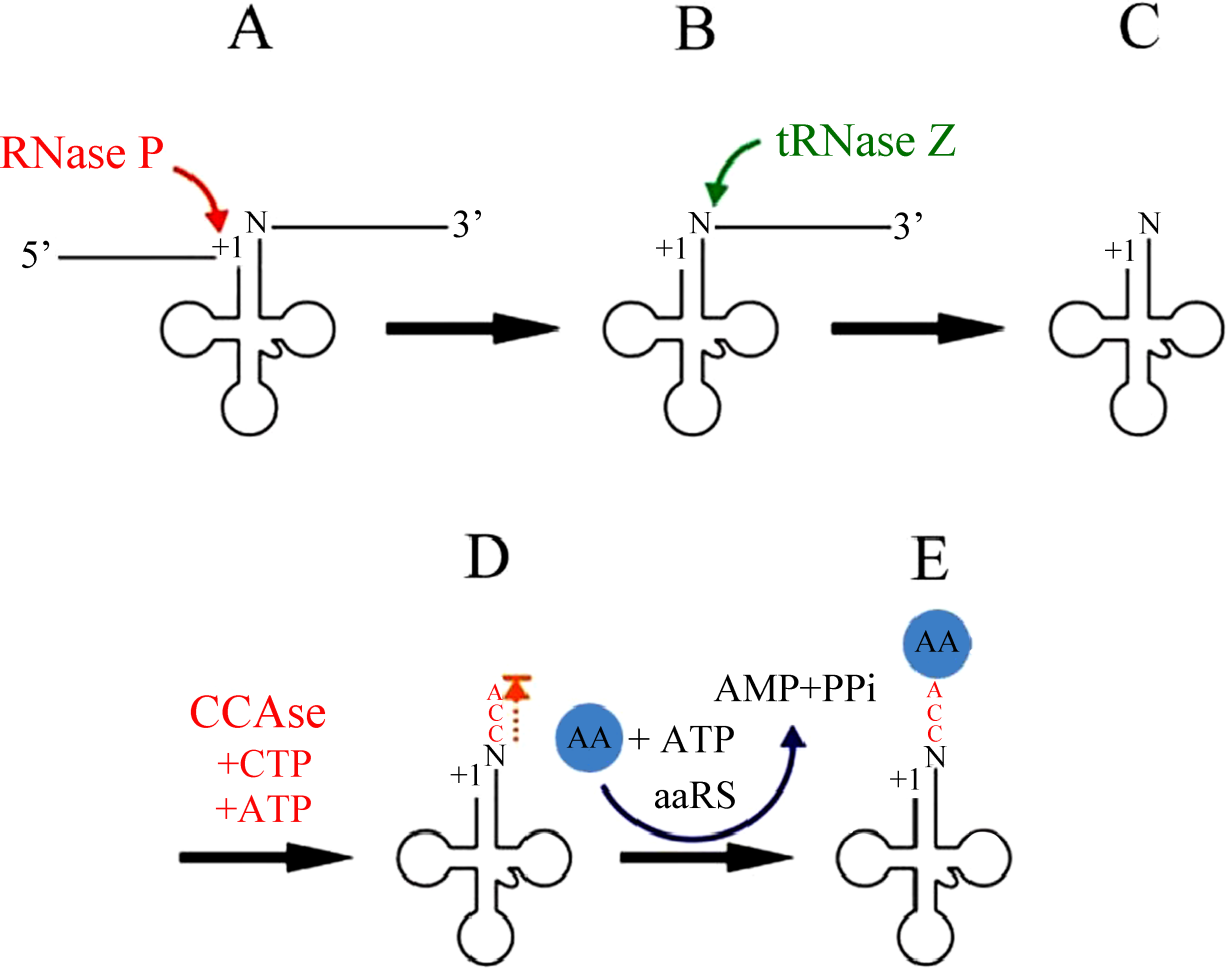
mt-tRNA 5′and 3′end processing pathway.

The excision of tRNAs from primary polycistronic mitochondrial transcripts is catalyzed by two specialized enzymes, RNase P and tRNase Z (Fig. 2). RNase P is an endonuclease that catalyzes the cleavage of the 5′leader sequence from pre-tRNA transcripts (*Rossmanith et al., 1995*). Human mitochondrial RNase P (mtRNase P) is a RNase P complex consisted of three proteins, called MRPP1; MRPP2 and MRPP3 (*Holzmann et al., 2008*). In fact, human mtRNase P cleaves a wide range of tRNA precursors in vitro (*Rossmanith, 1997; Rossmanith et al., 1995*), and its activity is detectable even in crude mitochondrial extracts and thereby appears to be relatively abundant (*Rossmanith et al., 1995*).

At the other end, 3′ trailers are removed by the endonuclease tRNase Z (Phizicky & Hopper, 2010; Hartmann et al., 2009; Maraia & Lamichhane, 2011). Both short and long forms of tRNase Z are present in eukaryotes, designated tRNase ZS (280 to 360 AAs) and tRNase ZL (750 to 930 AAs) respectively (*Ishii et al., 2005; Li de la Sierra-Gallay, Pellegrini & Condon, 2005*). The C-terminal part of tRNase ZL has sequence homology with tRNase ZS. However, in contrast to the single mechanism of 5′ leader removal, 3′ trailers can also be removed by one or more 3′ exoribonucleases (Rex1p, and perhaps others) (Copela et al., 2008; Ozanick et al., 2009). The 5′-before-3′ end processing appears to apply most clearly when tRNase Z is used for 3′ processing.

### tRNA post-transcriptional modifications

**Figure 3:**
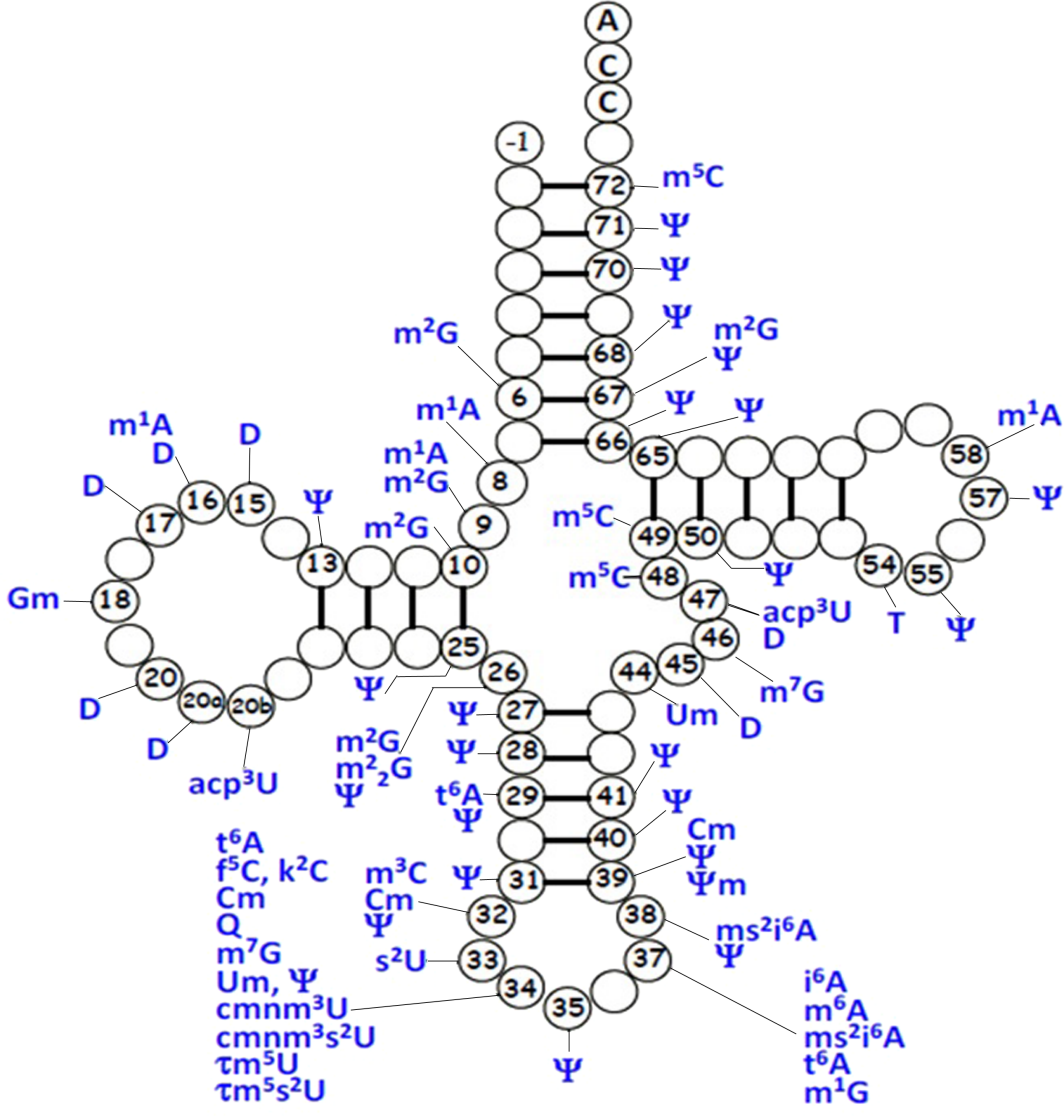
Distribution of post-transcriptional modifications in mt-tRNAs.

For mt-tRNA maturation, post-transcriptional modifications, together with the 5′and 3′nucleolytic excision from precursor RNAs are required. Certain modifications are necessary for maintenance of mt-tRNA structure and steady-state level, as well as ensuring the efficiency of protein synthesis during mitochondrial translation. Up to date, more than 30 modified mt-tRNA positions have been reported (*Suzuki, Nagao & Suzuki, 2011*) (Fig. 3). Modifications cluster occurs at two main regions of tRNA molecule: the structural core and the anticodon stem. Chemical modifications in the structural region are relatively simple, for instance, methylation, pseudouridylation and dihydrouridylation. Furthermore, modifications in the anticodon stem of mt-tRNAs include methylation and pseudouridylation, in several cases, with more complex additions, specially the modifications at positions 34 and 37 (El Yacoubi, Bailly & Crécy-Lagard, 2012). Four types of modified nucleotides were found at the wobble positions of 10 tRNA species that correspond to two codon sets. The modifications consisted of 5-formylcytidine at the wobble position of tRNA^Met^ (*Bilbille et al., 2011*), queuosine at the wobble positions of four tRNA^Tyr^, tRNA^His^, tRNA^Asn^ and tRNA^Asp^ (*Iwata-Reuyl, 2008*). In addition, five tRNAs were found to have taurine-containing uridines 5-taurinomethyluridine was identified in the tRNA^Leu(UUR)^ and tRNA^Trp^, and 5-taurinomethyl-2-thiouridine in tRNA^Lys^, tRNA^Glu^ and tRNA^Gln^ (*Nagao et al., 2009; Suzuki et al., 2002*).

### tRNA aminoacylation

Aminoacyl-tRNA synthetases (aaRSs), encoded by nuclear genes, play essential roles in protein synthesis. To start this process, aaRSs must catalyze the attachment of AAs to the corresponding tRNAs (*Yao & Fox, 2013*). This biochemical reaction requires the following steps: 1. AA+ATP →aminoacyl-AMP+PPi; 2. aminoacyl-AMP + tRNA →aminoacyl-tRNA+AMP. Today, nineteen species of aaRS genes were annotated in the human genome database (*Antonellis & Green, 2008*). Mammalian mitochondria had no enzyme corresponding to glutaminyl-tRNA synthetase (GlnRS) (*Nagao et al., 2009*). Mitochondrial LysRS and GlyRS were encoded by the same genes as the cytoplasmic LysRS and GlyRS, respectively, whereas the other aaRSs were encoded by genes different from the cytoplasmic ones (*Ling, Reynolds & Ibba, 2009*).

**Figure 4:**
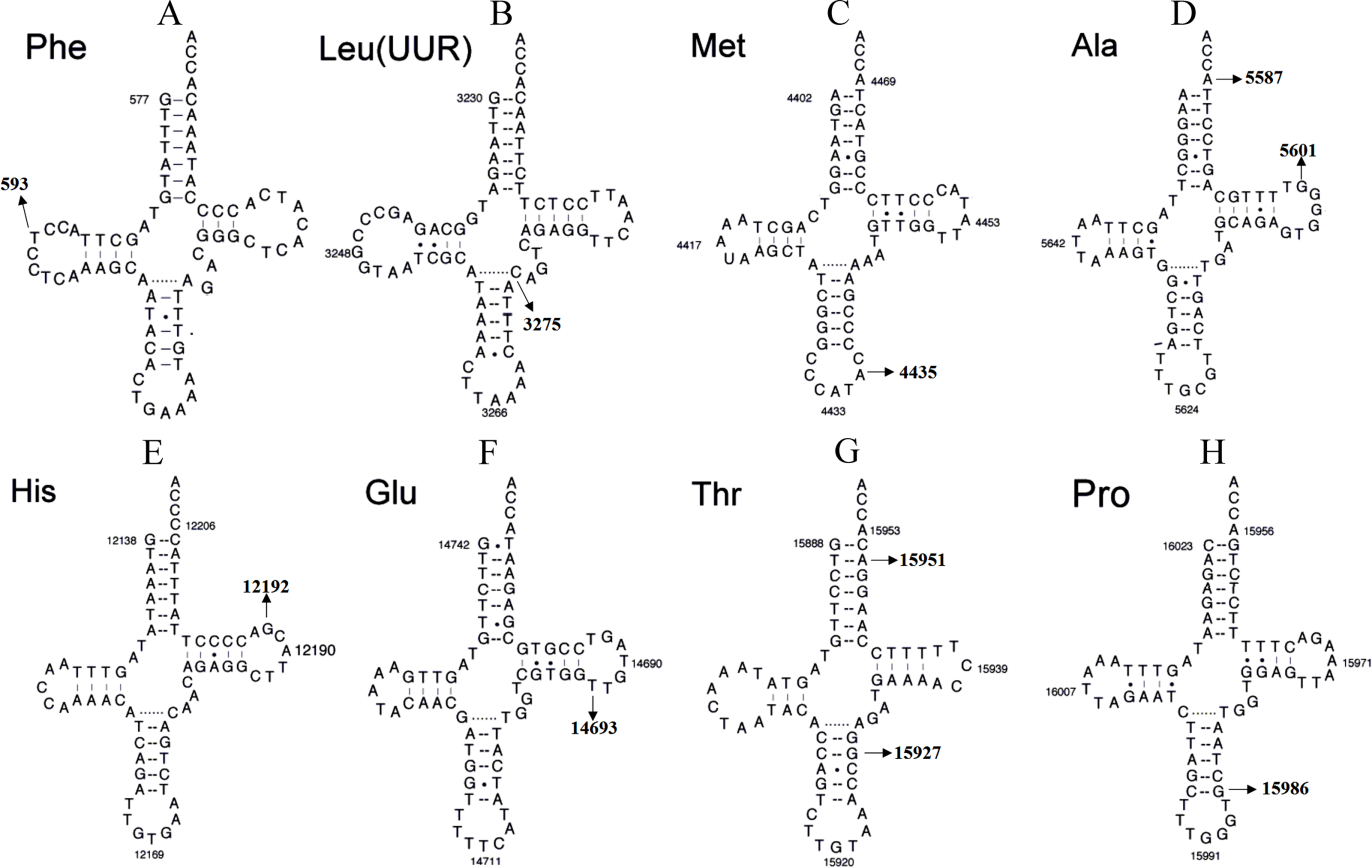
Secondary structure of (A) mt-tRNAPhe, (B) tRNALeu(UUR), (C) tRNAMet, (D) tRNAAla, (E) tRNAHis, (F) tRNAGlu, (G) tRNAThr and (H) tRNAPro.

### Secondary mt-tRNA variants

Although most LHON cases are caused by one of three pathogenic mtDNA mutations, no primary mutations are identified in a minority of LHON patients, these other homoplasmic mtDNA are considered as secondary variants that can be responsible for disease phenotype variation and different penetrance having synergistic effects with the primary mtDNA mutations (*Bosley & Abu-Amero, 2010; Zhang et al., 2009*) (Fig. 4 and Tables 1 and 2).

### mt-tRNA variants enhance the phenotypic expression of the primary mtDNA mutations

#### tRNA^**Phe**^ variant

According a recent experimental study, the 593T>C variant appears a high frequency in LHON patients (*Ji et al., 2008*). This variant occurs at the D-arm of tRNA^Phe^ anddecreases the free energy (*Zhang et al., 2011*). Moreover, the electrophoretic mobility of the tRNA ^Phe^gene with or without 593T>C transcribes confirm the change of secondary structure. Thus, the 593T>C variant may have a synergistic effect with the LHON-related 11778G>A mutation. By using lymphoblastoid cell lines derived from a Chinese family, an approximately ∼46% decrease in the steady-state level of tRNA^Phe^ was identified in mutant cell lines. Western blotting analysis showed an approximately 35% reduction in the levels of mitochondrial translation in mutant cell lines carrying the 593T>C variant (*Chen et al., 2017*). Interestingly, the 593T>C variant is suggested to increase the penetrance and expressivity of LHON-associated *ND6* 14484T>C mutation in one Chinese pedigree (*Man et al., 2020*)

**Table 1:** Characterization of 22 human mt-tRNAs.

tRNA Species	Starting	Ending	Length (bp)
tRNA^Phe^	577	647	71
tRNA^V al^	1,602	1,670	69
tRNA^Leu(UUR)^	3,230	3304	75
tRNA^Gln^	4,329	4,400	72
tRNA^Met^	4,402	4,469	68
tRNA^Trp^	5,512	5,579	68
tRNA^Ala^	5,587	5,655	69
tRNA^Asn^	5,657	5,729	73
tRNA^Cys^	5,761	5,826	66
tRNA^Tyr^	5,826	5,891	66
tRNA^Ser(UCN)^	7,446	7,514	69
tRNA^Asp^	75,18	7,585	68
tRNA^Lys^	8,295	8,364	70
tRNA^Gly^	9,991	10,058	68
tRNA^Arg^	10,405	10,469	65
tRNA^His^	12,138	12,206	69
tRNA^Ser(AGY )^	12,207	12,265	59
tRNA^Leu(CUN)^	12,266	12,336	71
tRNA^Glu^	14,674	14,742	69
tRNA^Thr^	15,888	15,953	66
tRNA^Pro^	15,956	16,023	68

**Table 2:** Summary of LHON-associated secondary mt-tRNA variants.

tRNA species	Allele	Position	Structural location	Homoplasmy or Heteroplasmy	Aberrant tRNA biology	References
tRNA^Phe^	593T>C	17	D-arm	Homoplasmy	Reduced expression of functional tRNA	Ji et al. (2008), Zhang et al. (2011)
tRNA^Leu(UUR)^	3275C>T	44	Variable region	Homoplasmy	Disrupt conserved base pairing	Garcia-Lozano et al. (2000), Ding et al. (2018)
tRNA^Met^	4435A>G	37	Anticodon stem	Homoplasmy	Introduce the m^1^G37 modification	Qu et al. (2006), Zhou et al. (2018)
tRNA^Ala^	5587T>C	73	Acceptor arm	Heteroplasmy	Affect the 3′end processing	Ji et al. (2017), Tang et al. (2010)
	5601C>T	59	T *ψ*C loop	Homoplasmy	Create conserved base pairing	Ding et al. (2020)
tRNA^His^	12192G>A	59	T *ψ*C loop	Homoplasmy	Disrupt conserved base pairing	Mimaki et al. (2003), Ding et al. (2019)
tRNA^Glu^	14693A>G	54	T *ψ*C loop	Homoplasmy	Defect in taurine modification	Tong et al. (2007), Zhang et al. (2010)
tRNA^Thr^	15927G>A	42	Anticodon stem	Homoplasmy	Disrupt conserved base pairing	Zhang et al. (2018), Jia et al. (2013)
	15951A>G	71	Acceptor arm	Homoplasmy	Disrupt conserved base pairing	Li et al. (2006), Lyu et al. (2019)
tRNA^Pro^	15986insG	39	Anticodon stem	Homoplasmy	Disrupt conserved base pairing	Jancic et al. (2020)

#### tRNA^**Met**^ variant

The 4435A>G variant affects a highly conserved adenosine at position 37, 3′ adjacent to the tRNA^Met^ anticodon, which is important for the fidelity of codon recognition and stabilization (*Lu et al., 2011*). This variant has been found to modulate the clinical expression of LHON-associated *ND4* 11778G>A mutation in a Chinese family (*Qu et al., 2006*). In fact, the 4435A>G variant introduces a tRNA methyltransferase 5 (TRMT5)-catalyzed m^1^G37 modification of tRNA^Met^. Functional analysis of cybrid cells harboring this variant indicated significantly decreased efficiency in aminoacylation and steady-state levels of tRNA^Met^, compared with findings in control cybrids (*Zhou et al., 2018*). An approximately 40% reduction in the level of tRNA^Met^ was observed in cells carrying the 4435A>G variant. The failure in tRNA metabolism, caused by the 4435A>G variant, led to an approximately 30% reduction in the rate of mitochondrial translation (*Liu et al., 2009*). These results indicate that the 4435A>G variant may lead to defects in mt-tRNA modification and enhance the phenotypic expression of LHON-related *ND4* 11778G>A mutation.

#### tRNA^**Ala**^ variant

According to recent report, the tRNA^Ala^5601C>T variant is associated with LHON (*(Ding et al., 2020)*). The homoplasmic 5601C>T variant has been reported in several LHON-affected pedigrees and one pedigree affected with hypertension (*Zhou et al., 2012; Zheng et al., 2018; Ding et al., 2020; Zheng et al., 2018*). This variant is located at very conserved nucleotide (position 59) in the TψC loop of tRNA^Ala^, and creates a novel Watson-Crick base-pairing (55T-59C). Bioinformatic analysis revealed that 5601C>T alters the secondary structure of tRNA^Ala^, thus, this variant contributes to the structural formation and stabilization of functional tRNA^Ala^ and leads to mitochondrial dysfunction caused by 11778G>A mutation.

#### tRNA^**His**^ variant

The 12192G>A variant, combined with the *ND4* 11778G>A mutation, has been reported in patients with both LHON and cardiomyopathy (*Mimaki et al., 2003*). Interestingly, the 12192G>A variant occurs 2-bp from the 3′ end of the TψC loop of tRNA^His^, which is highly conserved from various species (Pütz et al., 2007), and is believed to be involved in tertiary interaction between the TψC loop and the truncated D-arm (Ueda et al., 1992). Biochemical analysis of polymononuclear leukocytes (PMNs) which containing the 12192G>A variant showed a significant decrease in ATP production and an increased in ROS generation (Ding et al., 2019), suggesting that this polymorphism increases the penetrance and expressivity of LHON.

#### tRNA^**Glu**^ variant

The homoplasmic 14693A>G variant in the TψC loop of tRNA^Glu^ issuggested to modulate the phenotypic manifestation of LHON-associated *ND1* 3460G>A mutation in a Chinese pedigree (*Tong et al., 2007*). This variant has also been found in three LHON-affected families (*Zhang et al., 2010*). In fact, the 14693A>G variant is considered to be associated with mitochondrial encephalopathy, lactic acidosis, and stroke-like episodes (MELAS) (*Tzen et al., 2003*), PCOS (*Ding et al., 2017*), diabetes mellitus (*Tang et al., 2006*), and hearing loss (*Ding et al., 2009*). At the molecular level, the 14693A>G variant is located at very conserved nucleotide of tRNA^Glu^(conventional position 54), the nucleotide at that position in the TψC loop is often modified and contributes to the structural formation and stabilization of functional tRNAs (*Paris & Alfonzo, 2018*). Therefore, the change of structure of tRNAs due to this variant may lead to a failure in tRNA metabolism, which would in turn impair of mitochondrial translation.

#### tRNA^**Pro**^ variant

Recently, a novel mutation (15986insG) in mt-tRNA^Pro^ was identified in a Serbian family with LHON-associated 3460G>A mutation (Jancic et al., 2020). This insertion occurs at the anticodon stem of tRNA^Pro^, which disrupts a very conserved Watson-Crick base-pairing (31G-39C). In fact, tRNA^Ile^ 4298G>A occurring at the same position has been regarded as a pathogenic mutation associated with chronic progressive external ophthalmoplegia (CPEO) (Taylor et al., 1998). Thus, it can be speculated that 15986insG, which is similar to the 4298G>A mutation, may also lead to mitochondrial dysfunction that modulates the phenotypic expression of LHON-related 3460G>A mutation.

### Other reported mt-tRNA variants

#### tRNA^**Leu(UUR)**^ variant

According to a report by Garcia-Lozano et al. (2000), the 3275C>T variant in tRNA^Leu(UUR)^ contributes to the clinical expression of LHON and is associated with metabolic syndrome (MetS) and polycystic ovary syndrome (PCOS) (*Ding et al., 2018*). In fact, the homoplasmic 3275C>T variant disrupts a highly evolutionary conserved base-pairing (28A-46C) in the variable region of tRNA^Leu(UUR)^ (Salinas-Giegé, Giegé & Giegé, 2015), and bioinformatic analysis has revealed that the 3275C>T variant causes the thermodynamic change of tRNA^Leu(UUR)^. Moreover, patients with this variant have a lower level of mitochondrial membrane potential (MMP), ATP and mtDNA copy number, and higher ROS than the controls (*Ding et al., 2018*). Thus, the 3275C>T variant may lead to mitochondrial dysfunction, which is involved in the pathogenesis of LHON.

#### tRNA^**Ala**^ variant

The tRNA^Ala^5587T>C variant is reported to be associated with LHON according to a recent study (*Ji et al., 2017*). The heteroplasmic 5587T>C variant occurs at the end of the tRNA^Ala^ and may alter the tertiary structure of this tRNA (position 73), this nucleotide position is extremely conserved from bacteria to human mitochondria. Thus, it can be speculated that this variant influences the 3′end sequences of the acceptor arm of tRNA^Ala^, subsequently affecting the efficiency of protein translation. Furthermore, the 5587T>C variant has been shown to be associated with progressive unstable gait, dysarthria, hearing loss, muscle cramps and myalgia (*Tang et al., 2010; Crimi et al., 2002*).

#### tRNA^**Thr**^ variants

The tRNA^Thr^ gene is a “hot” spot for genetic variants associated with LHON, these variants included 15951A>G and 15927G>A (*Lyu et al., 2019*). Notably, the 15951A>G variant is localized at adjacent to 3′end (position 71) of tRNA ^Thr^, the adenine at this position is highly conserved from bacteria to human mitochondria (*Helm et al., 2000*).This nucleotide at position 71 of tRNAs has been shown to play an important role in the recognition by their cognate aaRS (*Florentz et al., 2003*). Furthermore, compared with controls, cybrid cells containing the 15951A>G variant showed an approximately ∼35% reduction in the level of tRNA^Thr^, the failure in tRNA metabolism resulting from this variant may lead to the impairment of mitochondrial translation (*Li et al., 2006*).

Moreover, the well-known 15927G>A variant disrupts a conservative base-pairing (28C-42G) in the anticodon stem of tRNA^Thr^. This variant was shown to be associated with an approximately ∼60% reduction in the level of tRNA^Thr^ in cybrid cells (*Zhang et al., 2018; Jia et al., 2013*). Additionally, western blot analysis showed the variable reductions of four mtDNA-encoded proteins in association with the variant, with especially marked decreases of *ND1* and *CYTB* (*Wang et al., 2008*). Furthermore, the 15927G>A variant was found to result in significantly reduced activities of Complexes I and III, as observed in cybrid cells (*Zhang et al., 2018*). Notably, the 15927G>A variant has also been reported to be associated with hearing loss (*Ding et al., 2019; Wang et al., 2008*) and coronary heart disease (*Jia et al., 2019*).

## Conclusions

Mitochondrial dysfunction and mtDNA genetic variants are linked to LHON. In previous studies, we noted that mainly LHON-associated pathogenic mtDNA mutations are located in genes encoding respiratory chain Complex I subunits. Moreover, secondary mt-tRNA variants may have synergistic effects on the clinical expression of LHON. In fact, mt-tRNA variants have structural and functional effects, including altering the tRNA secondary structure and the processing of tRNA precursors, reducing tRNA steady state level, and causing the defects in tRNA modifications. These events would exacerbate the mitochondrial dysfunction caused by the three primary mutations. Therefore, our findings are valuable for the further deepening our understanding of the pathophysiology and management of LHON.

## Additional Information and Declarations

### Data Availability

The following information was supplied regarding data availability: This is a literature review, therefore there is no raw data.
